# Departure of Prof. Flint for Europe

**Published:** 1854-03

**Authors:** 


### EDITORIAL DEPARTMENT.

*Departure of Prof. Flint for Europe.*—Our Senior Editor, after only a few days' sojourn with us on his return from Louisville, where his duties as a teacher have detained him since October last, left town on the morning of the 23d of February, intending to sail for Europe about the 1st of March.

During his absence, which will continue till the coming fall, we shall be furnished with letters from him of professional interest. A period of prolonged and exacting labor will render this vacation a grateful relaxation to him, and though our readers will be deprived of his personal supervision of the Journal, we doubt not that they will be compensated by the pleasure and profit of perusing his letters.

One word of personal interest, only, may be excused here. When we mounted the tripod, less than a year ago, we were entirely inexperienced in journalism. Circumstances have thrown upon us, for many months, its entire conduct. These circumstances will continue through the coming volume. With a more appreciative sense of the difficulties of medical journalism, and of the labor it involves, we do not find the task irksome. We have grown to a feeling of attachment to the Journal, its readers, and correspondents. Our best efforts will be directed to the maintenance of the good name of the Journal, and we may here be permitted to express the wish that many may be found to aid us in our task, by contributing to our pages the results of their experience. We know that a large modicum of the medical talent of the country is represented on our subscription list. Shall we not hear from it? H.

